# Supplementary material for: Complexation: An Interesting Pathway for Combining Two APIs at the Solid State
Source: Pharmaceutics. 2022 Sep 16;14(9):1960. doi: 10.3390/pharmaceutics14091960 (PMC9503796; doi:10.3390/pharmaceutics14091960)
Supplement: Supplementary file 1 [file pharmaceutics-14-01960-s001.zip › pharmaceutics-1878329-supplementary.pdf]

SUPPLEMENTARY MATERIALS

Complexation: An Interesting Pathway for Combining Two APIs at the Solid State

Fucheng Leng, Oleksii Shemchuk, Koen Robeyns and Tom Leyssens *

Institute of Condensed Matter and Nanosciences, Université catholique de Louvain, 1 Place Louis Pasteur, B-1348 Louvain-La-Neuve, Belgium.
Correspondence: tom.leyssens@uclouvain.be

Contents

1 Preparation method page 3

2 Crystallographic table page 4

3 Crystal structures page 6

3.1 Complexes of $Zn(IBU)_2$ page 6

3.2 Complexes of $Zn(PABA)_2$ page 8

3.3 Complexes of $Zn(ASP)_2$ page 10

4 Thermal analyses page 11

5 PXRD patterns page 13

5.1 The comparison of experimental and simulated PXRD patterns of $Zn(IBU)_2$-PD complexes. page 13

5.2 Mechanochemical screening results page 16

5.3 VT-PXRD page 22

6 Binary Cocrystal screen page 23

1. Preparation method

Crystal	Single crystal preparation	Bulk preparation
$Zn(IBU)_2(NC)$,	Zinc acetate, Ibu, NC dissolved by methanol in 1:2:1, then evaporation	Slurrying $Zn(IBU)_2$ and NC in 1:1 ratio with ethyl acetate as solvent
$Zn(PABA)_2(INC)_2 \cdot 0.5H_2O$	Zinc acetate, PABA, INC dissolved by methanol in 1:2:2, then evaporation	
$Zn(PABA)(Ac)(MN)_2 \cdot H_2O$	Zinc acetate, PABA, MN dissolved by methanol in 1:1:2, then evaporation	
$Zn(PABA)_2(NC)_2$	Zinc acetate, PABA, NC dissolved by methanol in 1:2:2, then evaporation	
$Zn(PABA)_2(INZ) \cdot CH_3OH$	Zinc acetate, PABA, INZ dissolved by methanol in 1:2:1, then evaporation	
$Zn(IBU)_2(INC)$	$Zn(IBU)_2$, INC dissolved by methanol in 1:1, then evaporation	Slurrying $Zn(IBU)_2$ and INC in 1:1 ratio with methanol as solvent
$Zn(ASP)_2(MN)_2$	$Zn(ASP)_2$, MN dissolved by methanol in 1:2, then evaporation	
$Zn(ASP)_2(INC)$	$Zn(ASP)_2$, INC dissolve by methanol in 1:1, then evaporation	
$Zn(PABA)_2(MN)_2 \cdot H_2O$	$Zn(PABA)_2$, MN dissolve by methanol in 1:2, then evaporation	
$Zn(IBU)_2(AMI)$	$Zn(IBU)_2$, AMI dissolved by methanol in 1:4, then evaporation and cool in fridge	Slurrying $Zn(IBU)_2$ and AMI in 1:1 ratio with isopropanol as solvent
$Zn(IBU)_2(INC)_2(H_2O)_2$	$Zn(IBU)_2$, INC dissolved by methanol/H_2O mixed solvent(7:3) in 1:2, then evaporation	Slurrying $Zn(IBU)_2$ and INC in 1:2 ratio with water as solvent
$Zn(IBU)_2(INZ)$	$Zn(IBU)_2$, INZ dissolved by methanol/H_2O mixed solvent(7:3) in 1:1, then evaporation	Slurrying $Zn(IBU)_2$ and INZ in 1:1 ratio with water as solvent
$Zn(PABA)_2(AMI)_2$	stoichiometric quantities of Zinc acetate, Ibu, NC dissolve in methanol than evaporation	

2. Crystallographic data.

Table S1a. Crystal data and structure refinement for the investigated structures. CCDC 2194913-2194925 contain the supplementary crystallographic data for this paper. These data can be obtained free of charge from The Cambridge Crystallographic Data Centre via www.ccdc.cam.ac.uk/structures.

**Identification code**	**$Zn(IBU)_2(NC)$**	**$Zn(IBU)_2(INC)_2(H_2O)_2$**	**$Zn(IBU)_2(INZ)$**	**$Zn(PABA)_2(INC)_2{\cdot}(H_2O)_{0.5}$**	**$Zn(IBU)_2(INC)$**	**$Zn(PABA)_2(AMI)_2$**	**$Zn_2(ASP)_4(INC)_2$**
**Empirical formula**	$C_{64} H_{80} N_4 O_{10} Zn_2$	$C_{38} H_{50} N_4 O_8 Zn$	$C_{32} H_{42} N_3 O_5 Zn$	$C_{52} H_{50} N_{12} O_{13} Zn_2$	$C_{38} H_{46} N_4 O_6 Zn$	$C_{62} H_{82} N_6 O_8 Zn_2$	$C_{30} H_{26} N_4 O_{10} Zn$
**Formula weight**	1196.06	756.19	614.05	1181.78	720.16	1170.07	667.92
**Temperature (K)**	297(2)	297(2)	297(2)	297(2)	297(2)	297(2)	297(2)
**Wavelength (Å)**	0.71073	0.71073	0.71073	0.71073	0.71073	0.71073	0.71073
**Crystal system**	Triclinic	Triclinic	Monoclinic	Monoclinic	Monoclinic	Triclinic	Monoclinic
**Space group**	*P*-1	*P*-1	$P2_1/c$	*I*2/*a*	*I*2/*a*	*P*-1	$P2_1/c$
**Unit cell dimensions (Å,°)**	a = 10.8461(8)	a = 5.5047(5)	a = 5.49695(15)	a = 22.7349(13)	a = 20.7691(13)	a = 10.7578(13)	a = 19.2090(10)
	b = 11.1730(14)	b = 10.9599(6)	b = 15.4850(5)	b = 11.6737(6)	b = 5.4155(3)	b = 11.2000(9)	b = 8.4481(6)
	c = 15.667(2)	c = 16.9517(16)	c = 36.8845(9)	c = 20.3594(11)	c = 33.352(2)	c = 14.613(2)	c = 19.0056(12)
	α= 109.991(12)	α= 72.152(7)	α= 90	α= 90	α= 90	α= 84.364(10)	α= 90
	β= 95.467(9)	β= 86.676(8)	β= 89.950(3)	β= 90.019(5)	β= 105.757(6)	β= 75.219(12)	β= 94.578(5)
	γ = 111.284(10)	γ = 89.379(6)	γ = 90	γ = 90	γ = 90	γ = 65.014(11)	γ = 90
**Volume ($Å^3$)**	1609.5(4)	971.83(14)	3139.61(15)	5403.4(5)	3610.3(4)	1543.0(4)	3074.4(3)
**Z**	1	1	4	4	4	1	4
**Density (calc.) (g/cm^3)**	1.234	1.292	1.299	1.453	1.325	1.259	1.443
**Absorption coeff. (mm^{-1})**	0.802	0.686	0.825	0.963	0.731	0.834	0.862
**F(000)**	632	400	1300	2440	1520	620	1376
**Crystal size (mm^3)**	0.50x 0.07 x 0.05	0.20x 0.10 x 0.06	0.30x 0.18 x 0.01	0.80x 0.10 x 0.04	0.15x 0.05 x 0.03	0.15x 0.15 x 0.10	0.50x 0.50 x 0.05
**Theta range for data collection (°)**	3.280 to 25.255	3.639 to 25.246	3.059 to 25.254	3.205 to 26.148	2.538 to 26.034	3.255 to 25.514	2.640 to 25.417
**Reflections collected**	19056	14375	5682	17451	12341	18613	5552
**Independent reflections**	5803 [R(int) = 0.0682]	3521 [R(int) = 0.0633]	5682 [R(int) = ?]	5357 [R(int) = 0.0415]	3559 [R(int) = 0.0480]	5740 [R(int) = 0.0560]	5552 [R(int) = ?]
**Completeness to θ=25.242° (%)**	99.7	99.8	99.6	99.6	99.8	99.7	99.8
**Absorption correction**	Semi-empirical from equivalents						
**Max. and min. transmission**	1.00000 and 0.46090	1.00000 and 0.87178	1.00000 and 0.07930	1.00000 and 0.87119	1.00000 and 0.88261	1.00000 and 0.84376	1.00000 and 0.72332
**Refinement method**	Full-matrix least-squares on F^2						
**Data / restraints / parameters**	5803 / 317 / 548	3521 / 127 / 278	5682 / 52 / 407	5357 / 1 / 360	3559 / 46 / 255	5740 / 197 / 450	5552 / 0 / 410
**Goodness-of-fit on F^2**	1.095	1.058	1.059	1.051	1.038	1.033	1.097
**Final R indices [I>2s(I)]**	R1 = 0.0636, wR2 = 0.1546	R1 = 0.0552, wR2 = 0.1423	R1 = 0.0359, wR2 = 0.1003	R1 = 0.0368, wR2 = 0.0959	R1 = 0.0439, wR2 = 0.0986	R1 = 0.0559, wR2 = 0.1429	R1 = 0.0640, wR2 = 0.1686

**R indices (all data)**	R1 = 0.0815, wR2 = 0.1645	R1 = 0.0639, wR2 = 0.1494	R1 = 0.0392, wR2 = 0.1028	R1 = 0.0454, wR2=0.1011	R1 = 0.0611, wR2 = 0.1055	R1 = 0.0699, wR2 = 0.1520	R1 = 0.0732, wR2 = 0.1753
**Δρ (max,min)(e.Å^{-3})**	1.113, -0.562	1.319, -0.434	0.338, -0.325	0.481, -0.238	0.375, -0.325	0.865, -0.817	1.101, -0.488

Table S1b. Crystal data and structure refinement for the investigated structures.

**Identification code**	**Zn(IBU)$_2$(AMI)**	**Zn(PABA)$_2$(NIC)$_2$**	**Zn(PABA)(Ac)(MN)$_2$·H$_2$O**	**Zn(PABA)$_2$(MN)$_2$·H$_2$O**	**Zn(PABA)$_2$(INZ)·CH$_3$OH**	**Zn(PABA)$_2$(AMI)$_2$**
**Empirical formula**	$C_{62}H_{82}N_6O_8Zn_2$	$C_{26}H_{24}N_6O_6Zn$	$C_{32}H_{32}N_4O_{12}Zn_2$	$C_{28}H_{28}N_4O_9Zn$	$C_{21}H_{23}N_5O_6Zn$	$C_{24}H_{26}N_8O_4Zn$
**Formula weight**	1170.07	581.88	795.35	629.91	506.81	555.90
**Temperature (K)**	297(2)	297(2)	297(2)	297(2)	297(2)	297(2)
**Wavelength (Å)**	0.71073	0.71073	0.71073	0.71073	0.71073	0.71073
**Crystal system**	Triclinic	Monoclinic	Monoclinic	Monoclinic	Triclinic	Monoclinic
**Space group**	*P*-1	*C*2/*c*	*P*2$_1$/*n*	*P*2/*c*	*P*-1	*P*2$_1$/*c*
**Unit cell dimensions (Å,°)**	a = 10.7578(13)	a = 27.119(2)	a = 10.5805(10)	a = 15.2611(14)	a = 9.6899(17)	a = 10.0747(10)
	b = 11.2000(9)	b = 5.8028(5)	b = 12.6078(14)	b = 16.2486(11)	b = 10.4485(7)	b = 19.0081(18)
	c = 14.613(2)	c = 16.6168(15)	c = 13.0496(15)	c = 11.2437(7)	c = 12.2345(16)	c = 13.0252(13)
	α= 84.364(10)	α= 90	α= 90	α= 90	α= 70.702(10)	α= 90
	β= 75.219(12)	β= 97.624(8)	β= 107.058(12)	β= 103.123(8)	β= 88.709(12)	β= 103.795(11)
	γ = 65.014(11)	γ = 90	γ = 90	γ = 90	γ = 73.166(11)	γ = 90
**Volume (Å^3)**	1543.0(4)	2591.8(4)	1664.2(3)	2715.3(4)	1115.3(3)	2422.4(4)
**Z**	1	4	2	4	2	4
**Density (calc.) (g/cm^3)**	1.259	1.491	1.587	1.541	1.509	1.524
**Absorption coeff. (mm^{-1})**	0.834	1.001	1.511	0.968	1.149	1.063
**F(000)**	620	1200	816	1304	524	1152
**Crystal size (mm^3)**	0.15x 0.15 x 0.10	0.30x 0.10 x 0.08	0.30x 0.30 x 0.25	0.30x 0.28 x 0.10	0.50x 0.10 x 0.04	0.20x 0.20 x 0.15
**Theta range for data collection (°)**	3.255 to 25.514	3.068 to 26.195	2.721 to 26.206	2.398 to 25.772	2.586 to 26.185	2.681 to 26.324
**Reflections collected**	18613	8735	9950	7886	15465	15722
**Independent reflections**	5740 [R(int) = 0.0560]	2582 [R(int) = 0.0448]	3308 [R(int) = 0.0271]	7886 [R(int) = ?]	4324 [R(int) = 0.0269]	4878 [R(int) = 0.0371]
**Completeness to θ=25.242° (%)**	99.7	99.7	99.6	99.7	97.7	99.7
**Absorption correction**	Semi-empirical from equivalents					
**Max. and min. transmission**	1.00000 and 0.84376	1.00000 and 0.88234	1.00000 and 0.94940	1.00000 and 0.81152	1.00000 and 0.75799	1.00000 and 0.44842
**Refinement method**	Full-matrix least-squares on F^2					
**Data / restraints / parameters**	5740 / 197 / 450	2582 / 0 / 177	3308 / 0 / 231	7886 / 0 / 385	4324 / 0 / 300	4878 / 0 / 334
**Goodness-of-fit on F^2**	1.033	1.072	1.034	1.050	1.046	1.026
**Final R indices [I>2s(I)]**	R1 = 0.0559, wR2 = 0.1429	R1 = 0.0350, wR2 = 0.0826	R1 = 0.0290, wR2 = 0.0759	R1 = 0.0602, wR2 = 0.1538	R1 = 0.0370, wR2 = 0.1020	R1 = 0.0405, wR2 = 0.0989
**R indices (all data)**	R1 = 0.0699, wR2 = 0.1520	R1 = 0.0420, wR2 = 0.0857	R1 = 0.0326, wR2 = 0.0781	R1 = 0.0750, wR2 = 0.1608	R1 = 0.0385, wR2 = 0.1034	R1 = 0.0504, wR2 = 0.1049
**Δρ (max,min)(e.Å^{-3})**	0.865, -0.817	0.284, -0.181	0.338, -0.244	0.623, -0.585	0.750, -0.532	0.461, -0.411

3. Crystal structures.

3.1 Complexes of $Zn(IBU)_2$

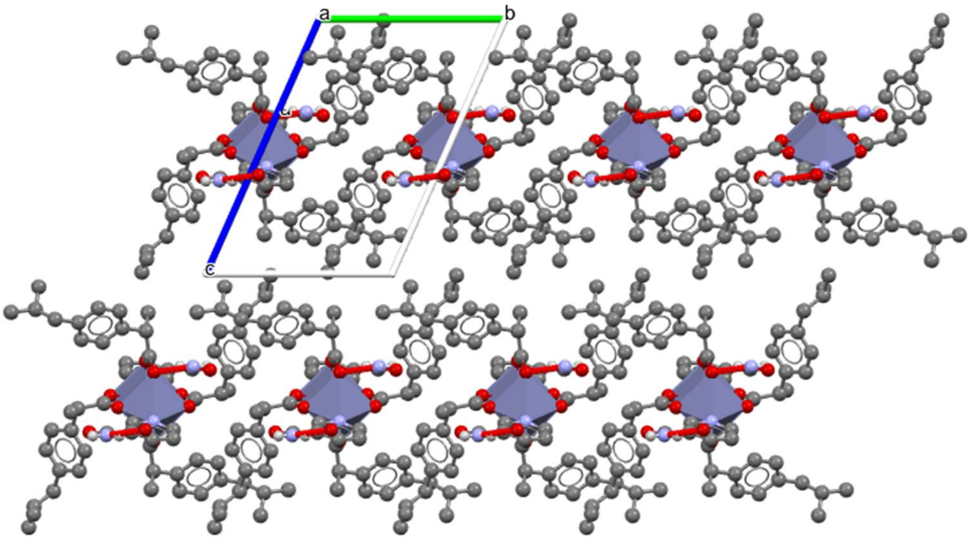

Figure S1. Crystal packing of $Zn(IBU)_2(NC)$; view along crystallographic a-axis. H_{CH} are omitted for clarity.

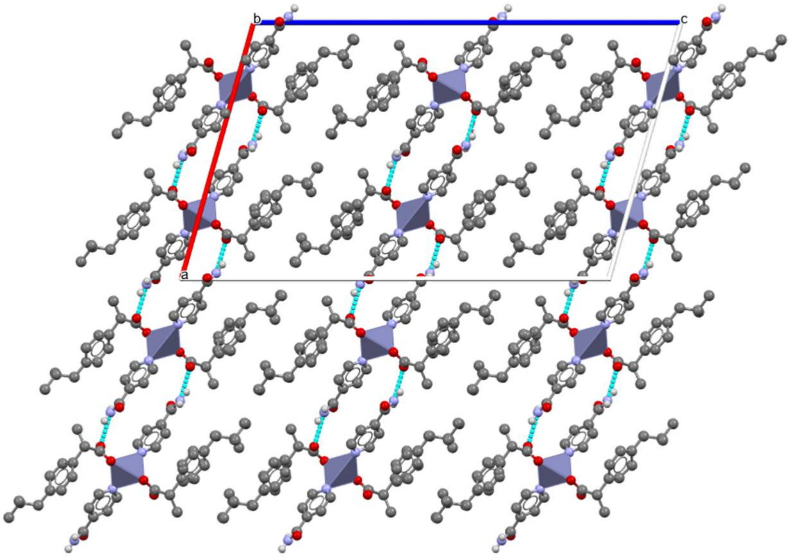

Figure S2. Crystal packing of $Zn(IBU)_2(INC)_2$; view along crystallographic b-axis. H_{CH} are omitted for clarity.

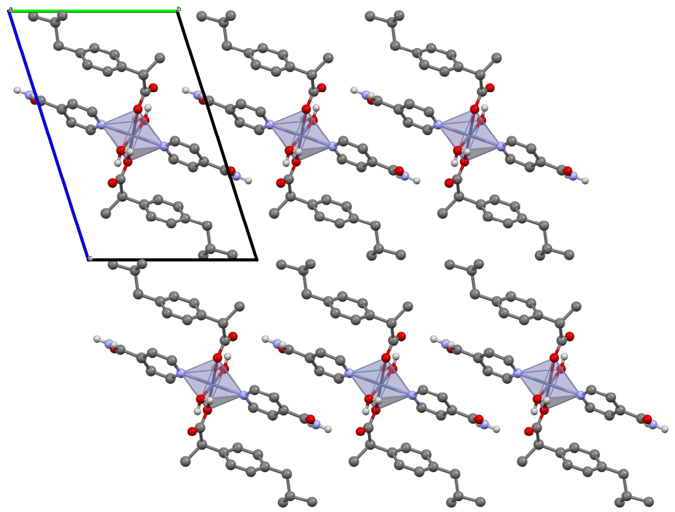

Figure S3. Crystal packing of $Zn(IBU)_2(INC)_2(H_2O)_2$; view along crystallographic b-axis. H_{CH} omitted for clarity.

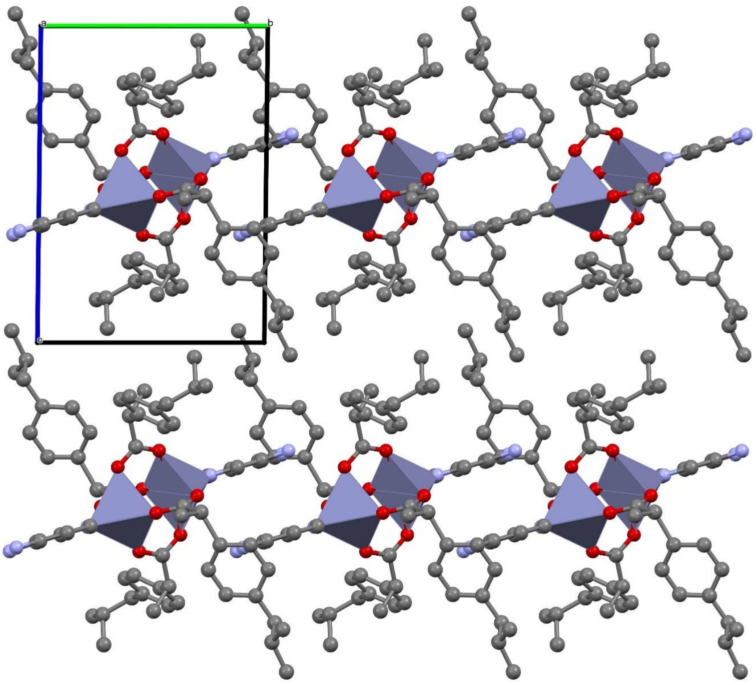

Figure S4. Crystal packing of $Zn(IBU)_2(AMI)$; view down crystallographic a-axis. Hydrogen atoms omitted for clarity.

3.2 Complexes of $Zn(PABA)_2$

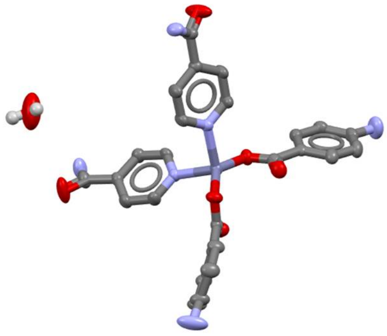

Figure S5. Tetragonal coordination structure in $Zn(PABA)_2(INC)_2 \cdot 0.5H_2O$. Hydrogen atoms are omitted for clarity.

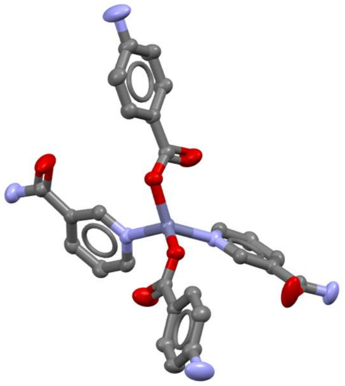

Figure S6. Tetragonal coordination structure in $Zn(PABA)_2(NC)_2$. Hydrogen atoms are omitted for clarity.

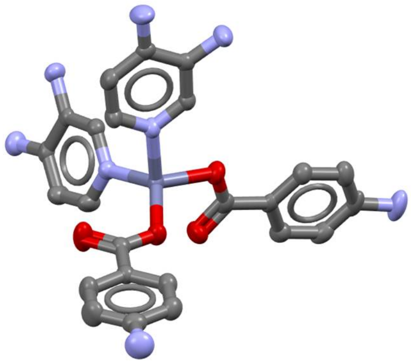

Figure S7. Tetragonal coordination structure in $Zn(PABA)_2(AMI)_2$. Hydrogen atoms are omitted for clarity.

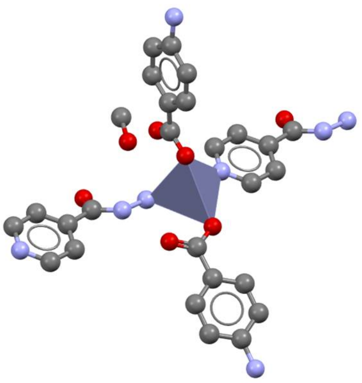

Figure S8. Tetragonal coordination structure in $Zn(PABA)_2(INZ)\cdot CH_3OH$. Hydrogen atoms omitted for clarity.

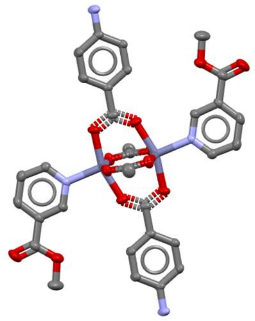

Figure S9. Paddle-wheel coordination structure in $Zn(PABA)(Ac)(MN)\cdot H_2O$. Hydrogen atoms are omitted for clarity.

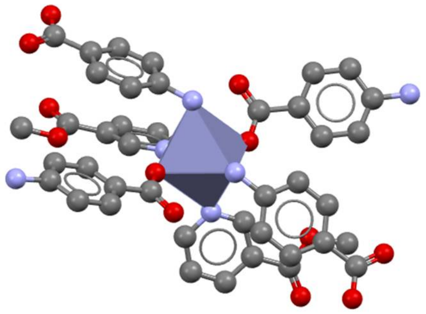

Figure S10. Octahedral coordination structure in $Zn(PABA)_2(MN)_2\cdot H_2O$. Hydrogen atoms omitted for clarity.

3.3 Complexes of $Zn(ASP)_2$

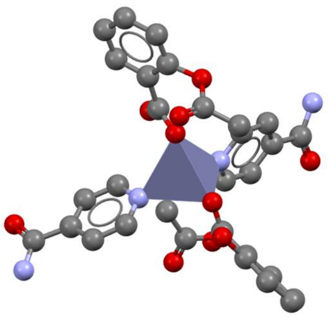

Figure S11. tetragonal coordination structure in Zn $(ASP)_2(INC)_2$. Hydrogen atoms omitted for clarity.

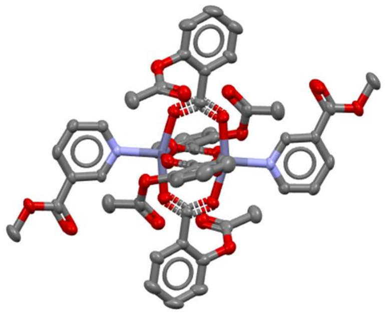

Figure S12. Paddle-wheel coordination structure in $Zn(ASP)_2(MN)$. Hydrogen atoms omitted for clarity.

3. Thermal analyses.

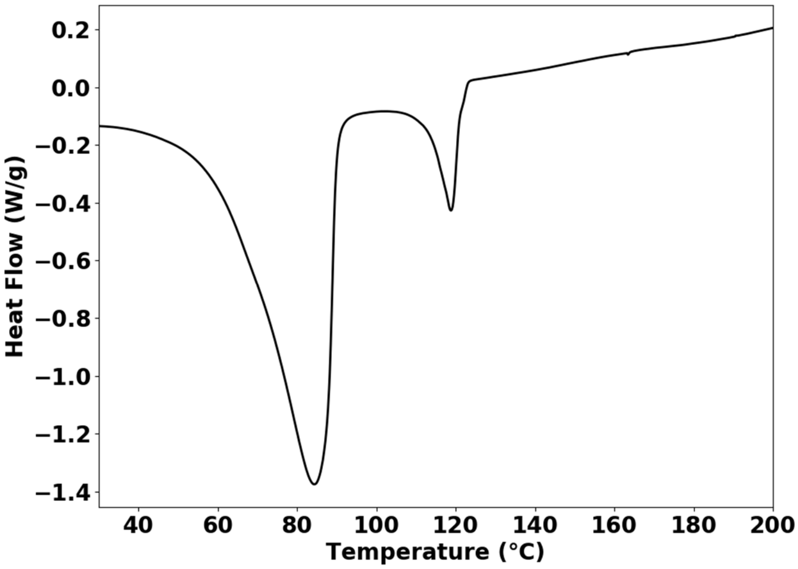

Figure S13. DSC thermogram of $Zn(IBU)_2(H_2O)_2$.

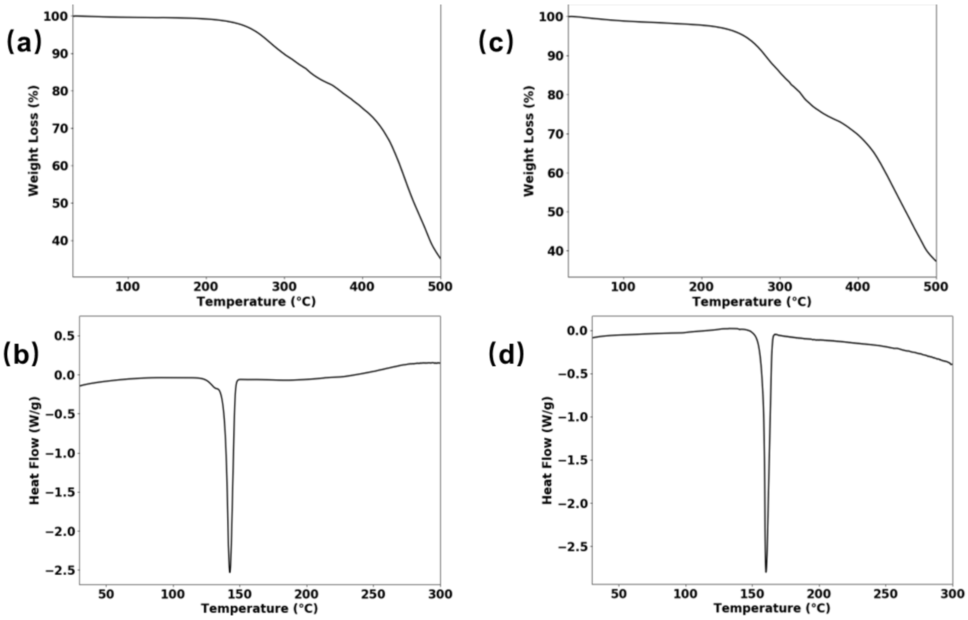

Figure S14 TGA and DSC thermograms of $Zn(IBU)_2(AMI)$ (**a** and **b**) and of $Zn(IBU)_2(AMI)_2$ (**c** and **d**).

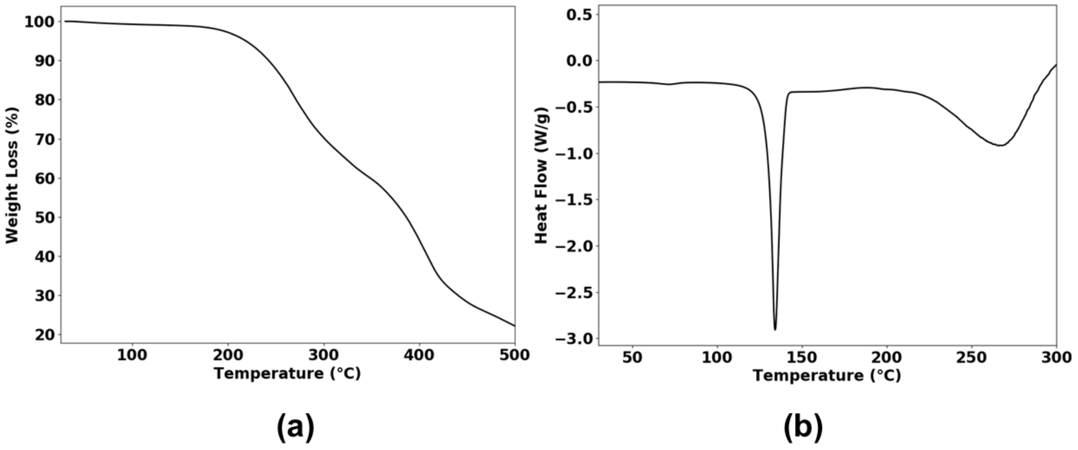

Figure S15. TGA (**a**) and DSC (**b**) thermograms of Zn(IBU)2(NC).

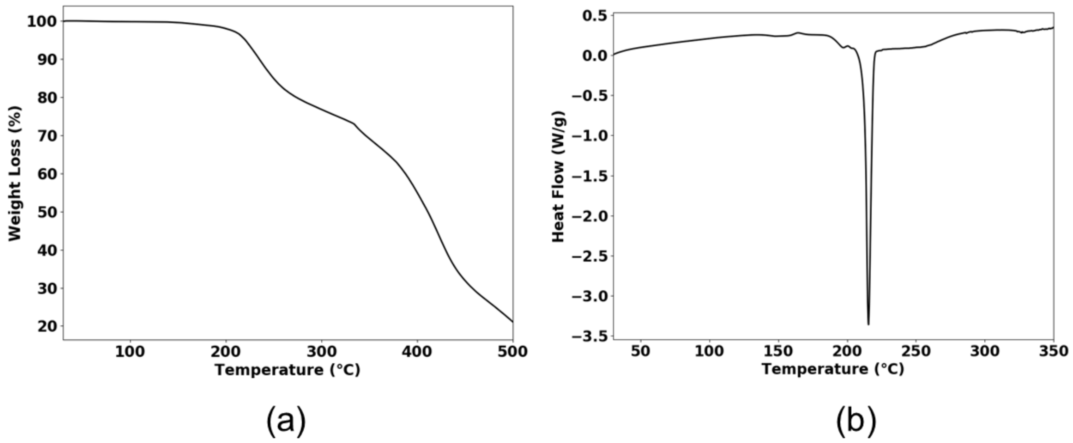

Figure S16. TGA (**a**) and DSC (**b**) thermograms of Zn(IBU)2(INZ).

4. PXRD patterns

4.1 The comparison of experimental and simulated PXRD patterns of Zn(IBU)₂-PD complexes.

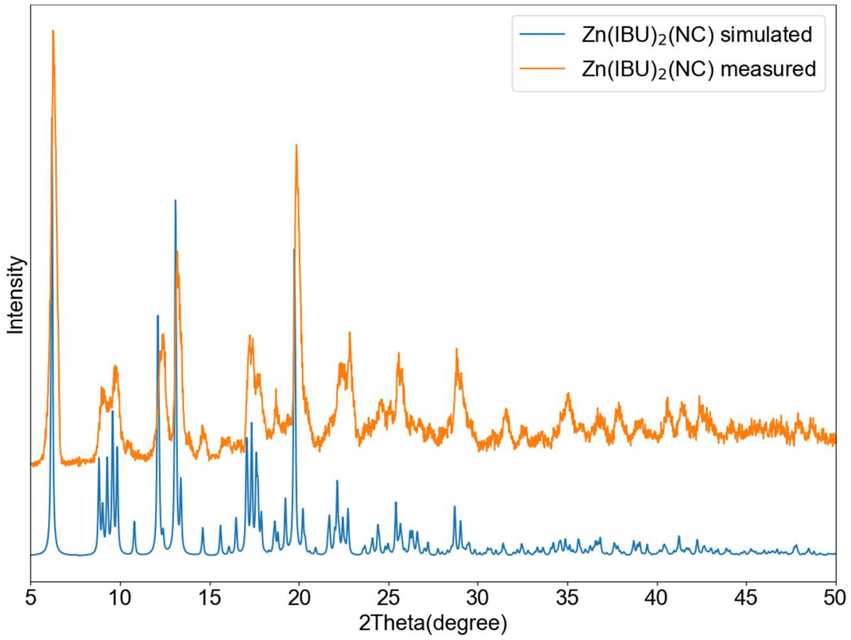

Figure S17. Comparison between the experimental (obtained by slurry in acetonitrile) and calculated PXRD patterns for $Zn(IBU)_2(NC)$.

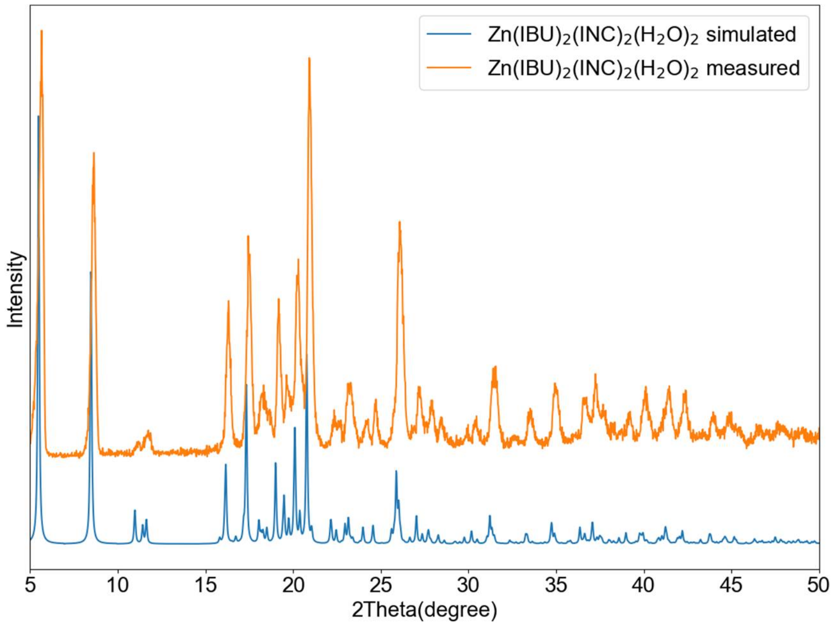

Figure S18. Comparison between the experimental (obtained by slurry in H_2O) and calculated PXRD patterns for $Zn(IBU)_2(INC)_2(H_2O)_2$.

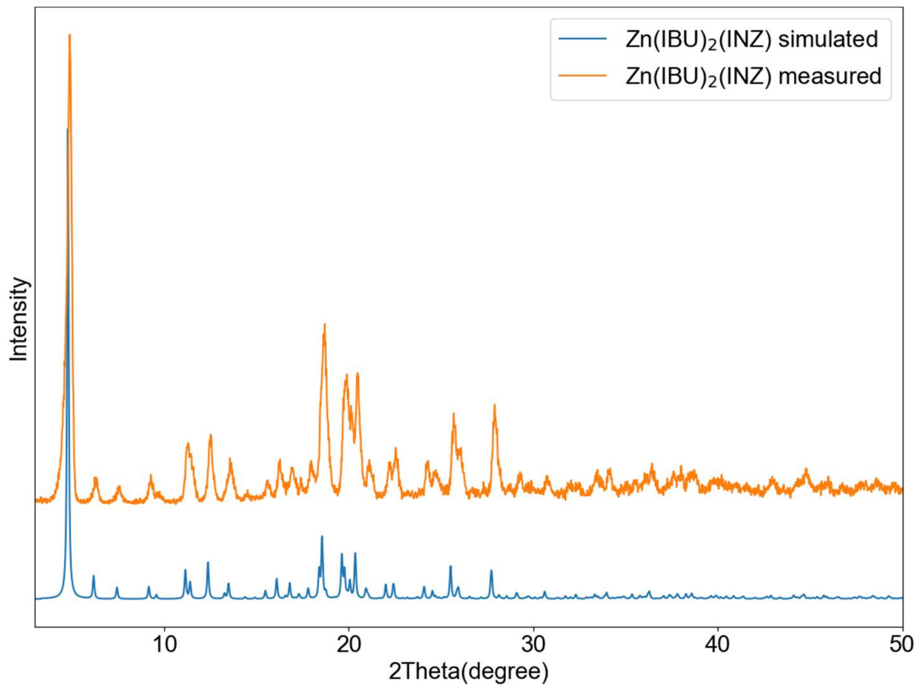

Figure S19. Comparison between the experimental (obtained by slurry in H_2O) and calculated PXRD patterns for $Zn(IBU)_2(INZ)$.

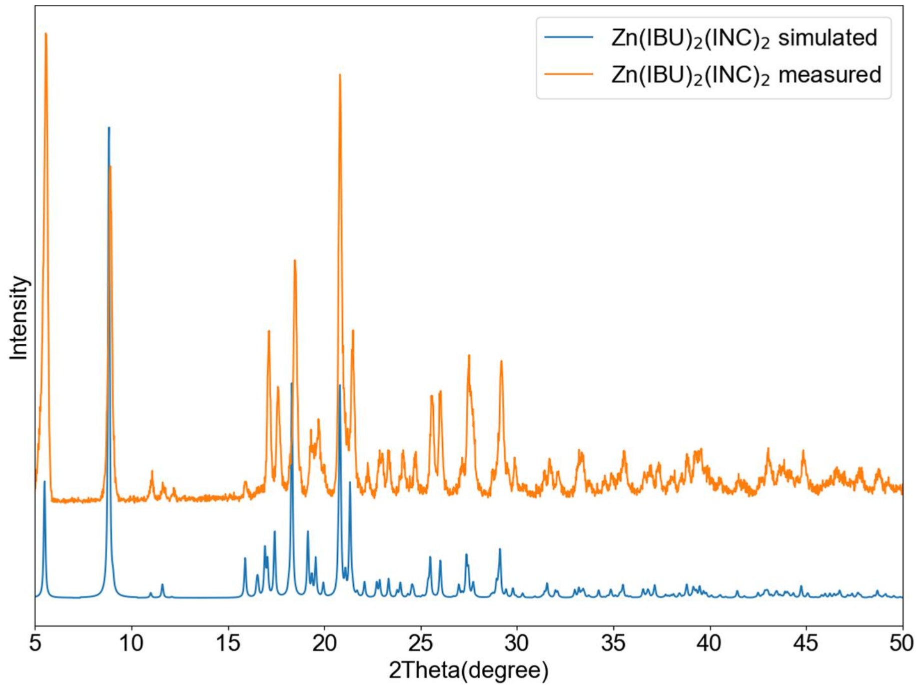

Figure S20. Comparison between the experimental (obtained by slurry in H_2O) and simulated PXRD patterns for $Zn(IBU)_2(INC)_2$.

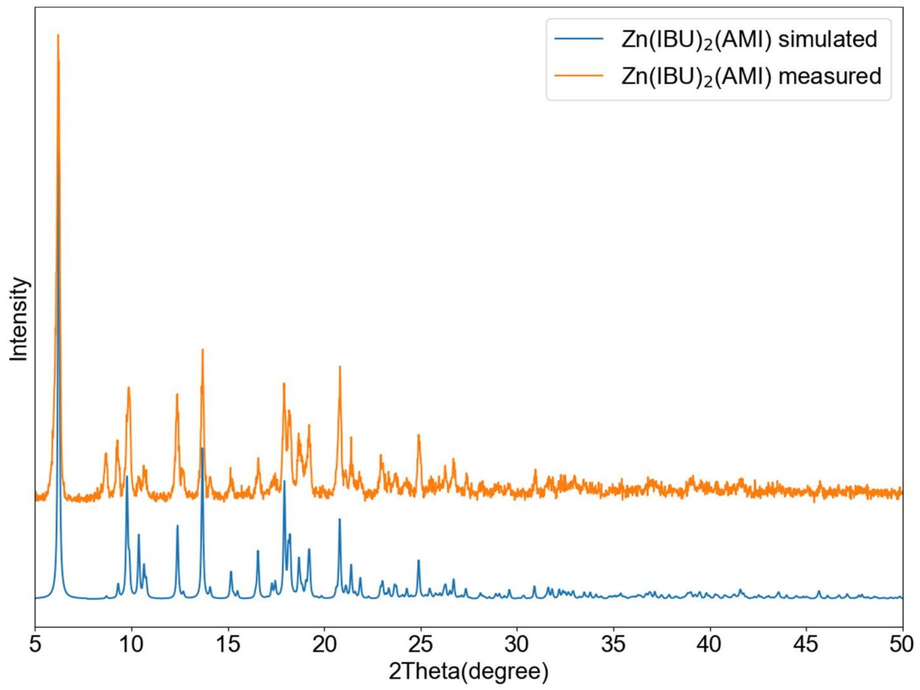

Figure S21. Comparison between the experimental (obtained by slurry in H_2O) and simulated PXRD patterns for $Zn(IBU)_2(AMI)$.

4.2 Mechanochemical screening results

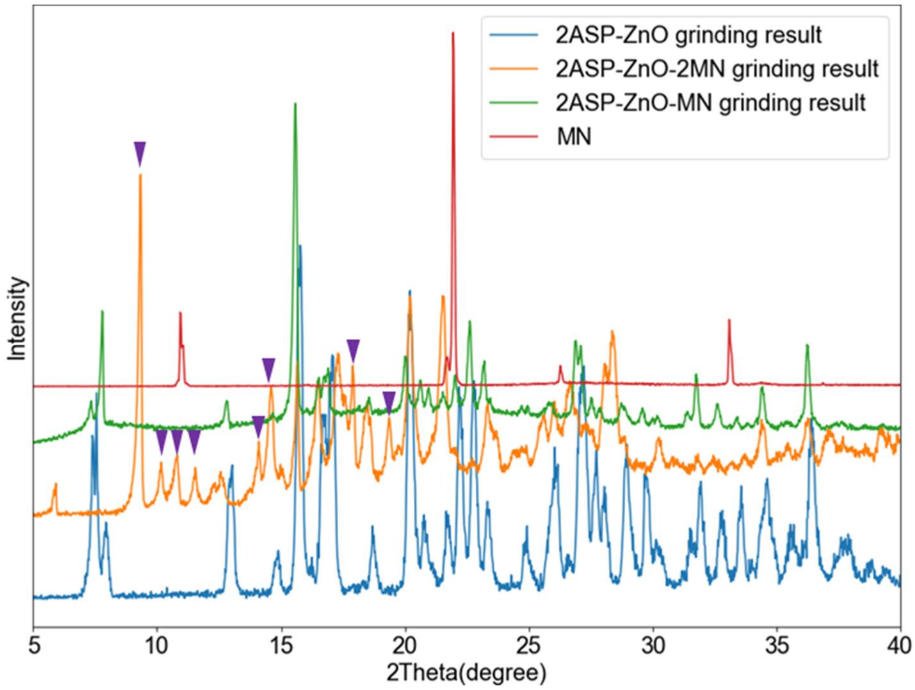

Figure S22. Comparison of PXRD patterns for MN (red), ASP-ZnO grinding result in 2:1 (blue) as well as ASP-ZnO-MN three-component grinding results in 2:1:1 (green) and 2:1:2 (orange) ratios.

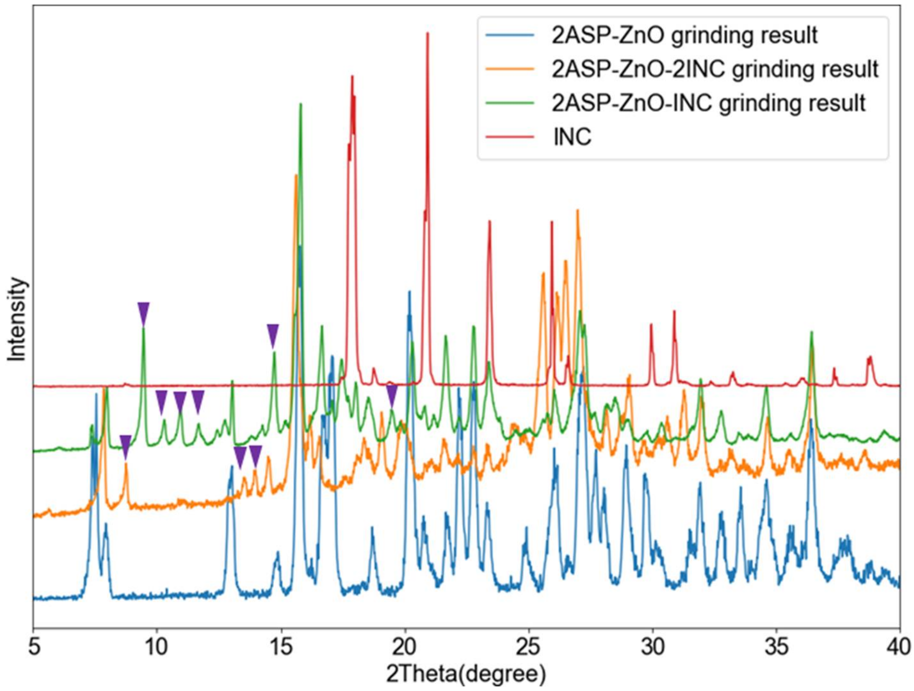

Figure S23. Comparison of PXRD patterns for INC (red), ASP-ZnO grinding result in 2:1 (blue) as well as ASP-ZnO-INC three-component grinding results in 2:1:1 (green) and 2:1:2 (orange) ratios.

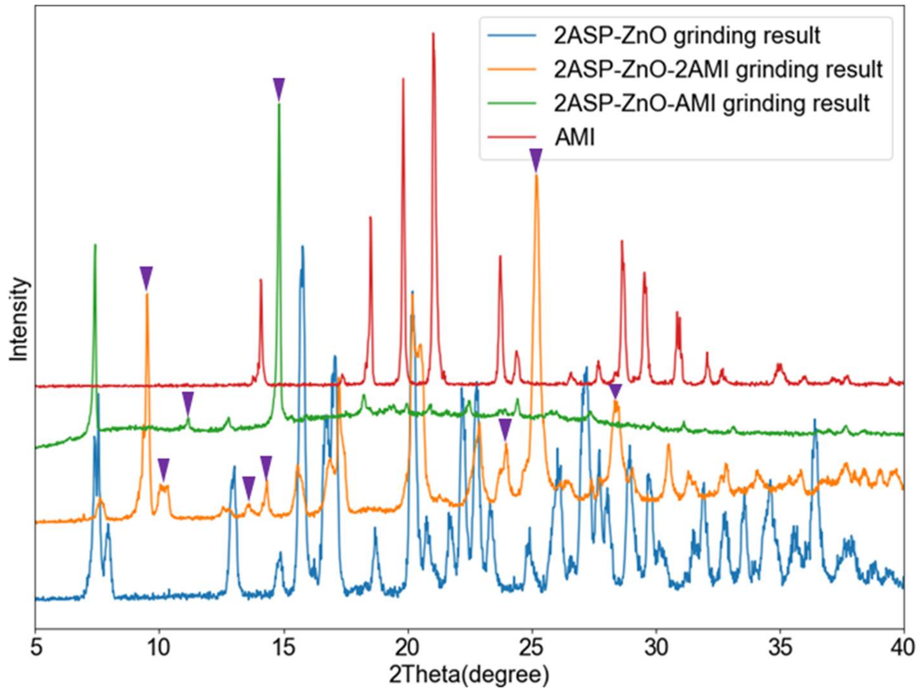

Figure S24. Comparison of PXRD patterns for AMI (red), ASP-ZnO grinding result in 2:1 (blue) and ASP-ZnO-AMI three-component grinding results in 2:1:1 (green) and 2:1:2 (orange) ratios.

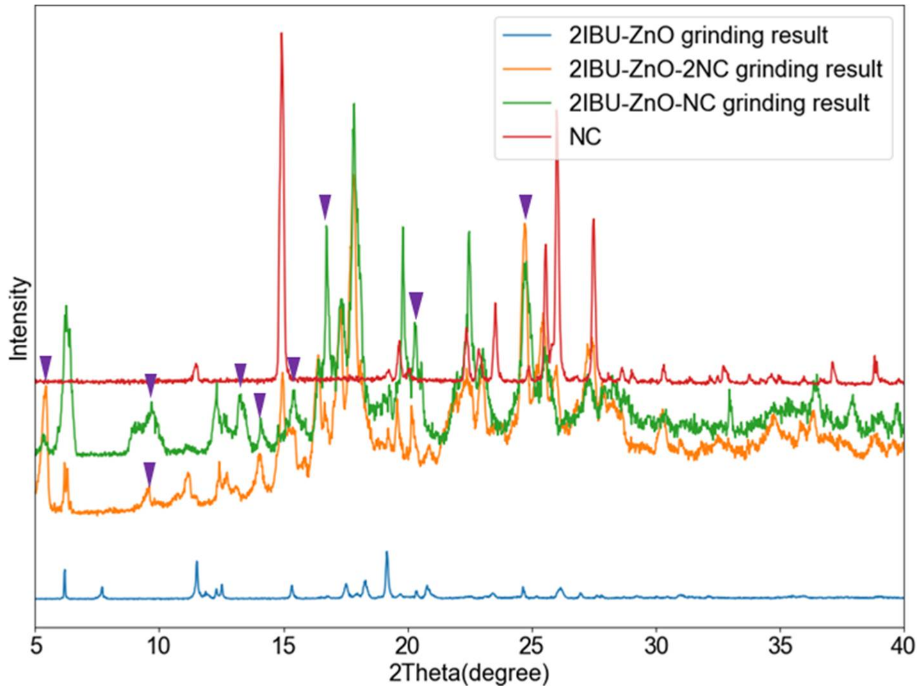

Figure S25. Comparison of PXRD patterns for NC (red), IBU-ZnO grinding result in 2:1 (blue) and IBU-ZnO-NC three-component grinding results in 2:1:1 (green) and 2:1:2 (orange) ratios.

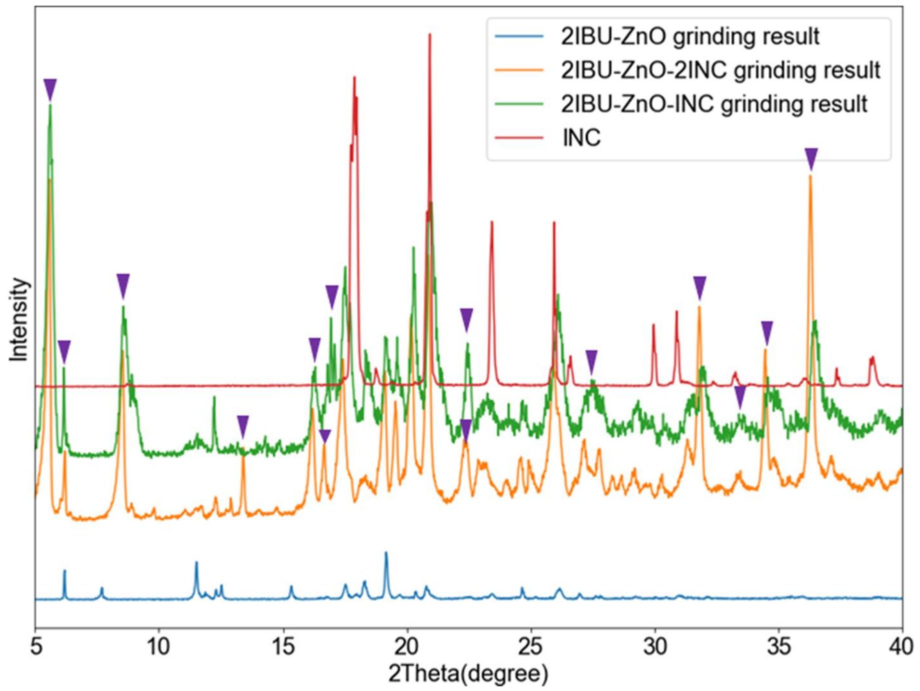

Figure S26. Comparison of PXRD patterns for INC (red), IBU-ZnO grinding result in 2:1 (blue) and IBU-ZnO-INC three-component grinding results in 2:1:1 (green) and 2:1:2 (orange) ratios.

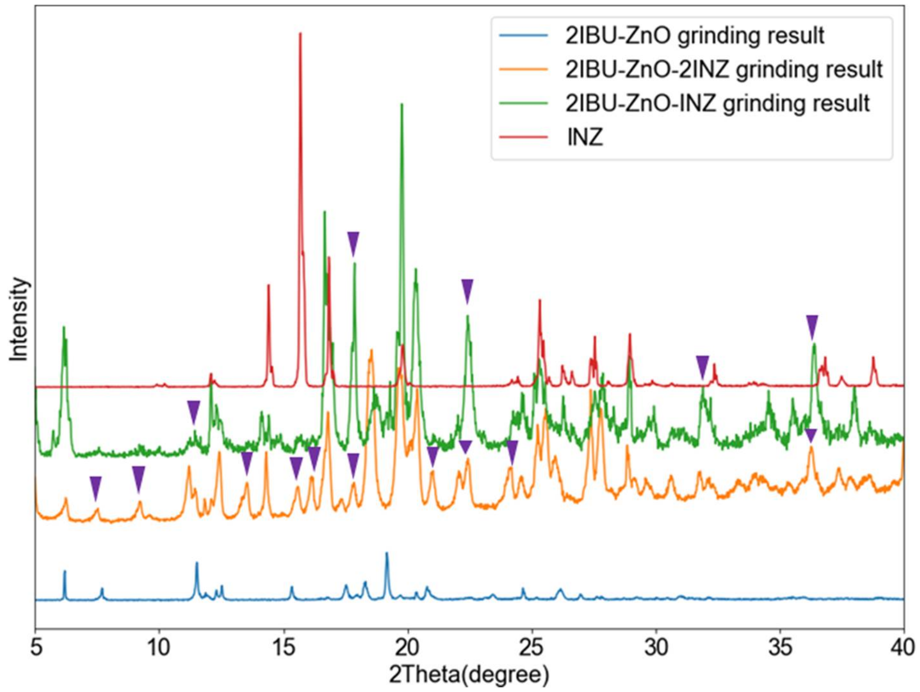

Figure S27. Comparison of PXRD patterns for INZ (red), IBU-ZnO grinding result in 2:1 (blue) and IBU-ZnO-INZ three-component grinding results in 2:1:1 (green) and 2:1:2 (orange) ratios.

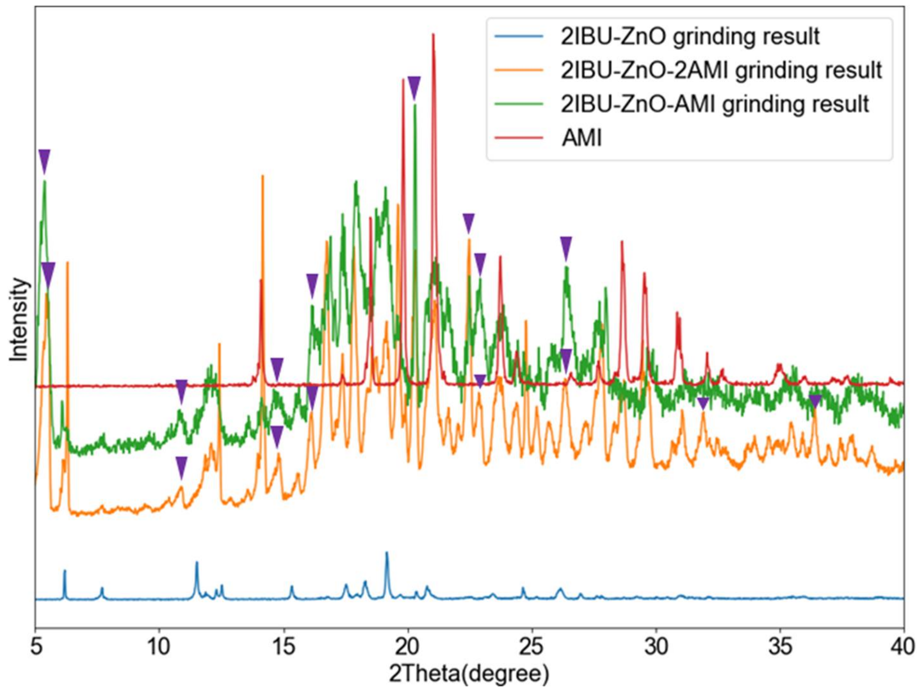

Figure S28. Comparison of PXRD patterns for AMI (red), IBU-ZnO grinding result in 2:1 (blue) and IBU-ZnO-AMI three-component grinding results in 2:1:1 (green) and 2:1:2 (orange) ratios.

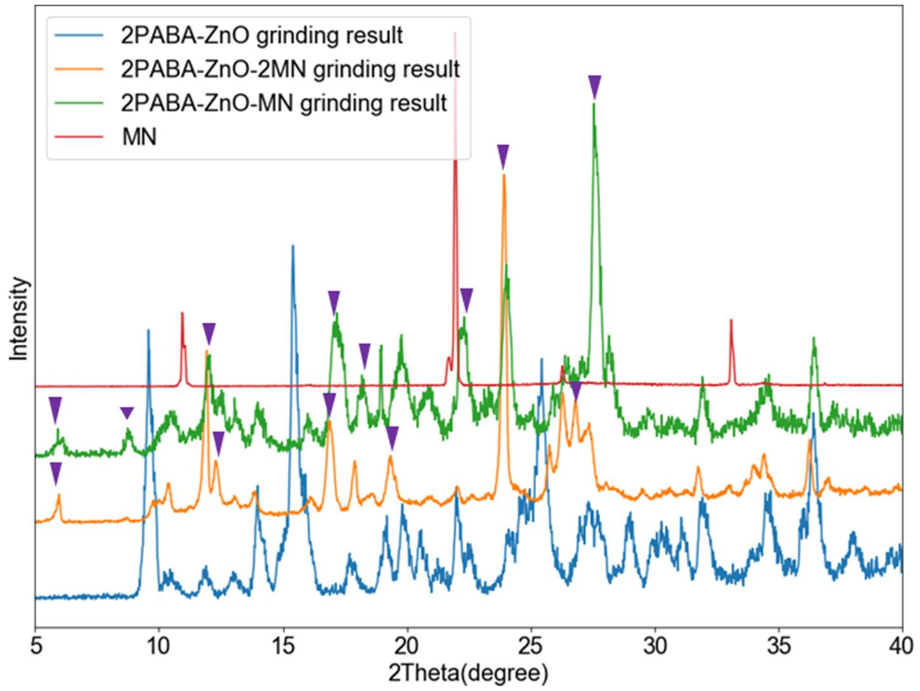

Figure S29. Comparison of PXRD patterns for MN (red), PABA-ZnO grinding result in 2:1 (blue) and PABA-ZnO-MN three-component grinding results in 2:1:1 (green) and 2:1:2 (orange) ratios.

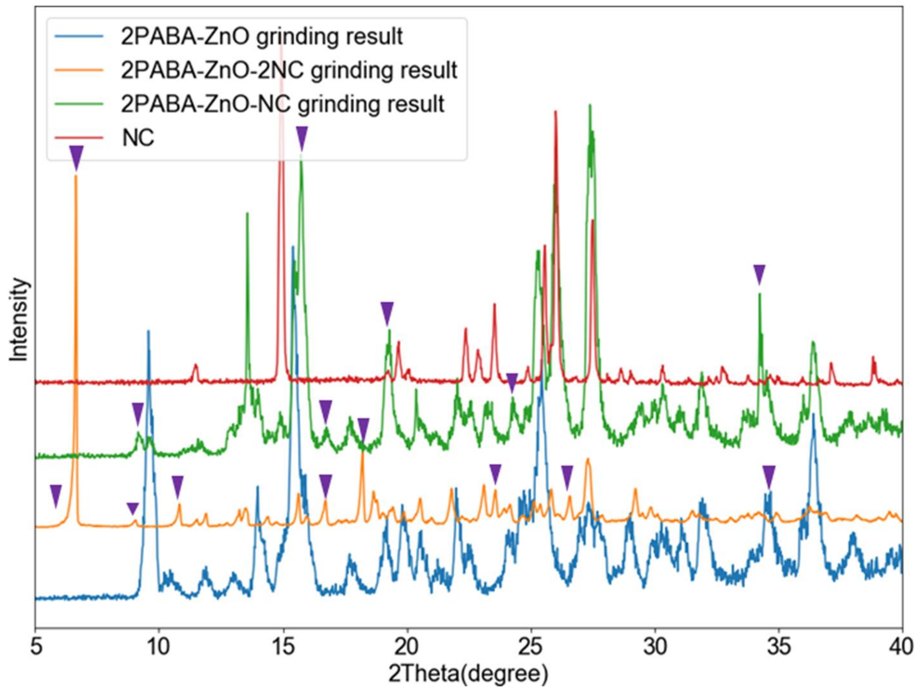

Figure S30. Comparison of PXRD patterns for NC (red), PABA-ZnO grinding result in 2:1 (blue) as well as PABA-ZnO-NC three-component grinding results in 2:1:1 (green) and 2:1:2 (orange) ratios.

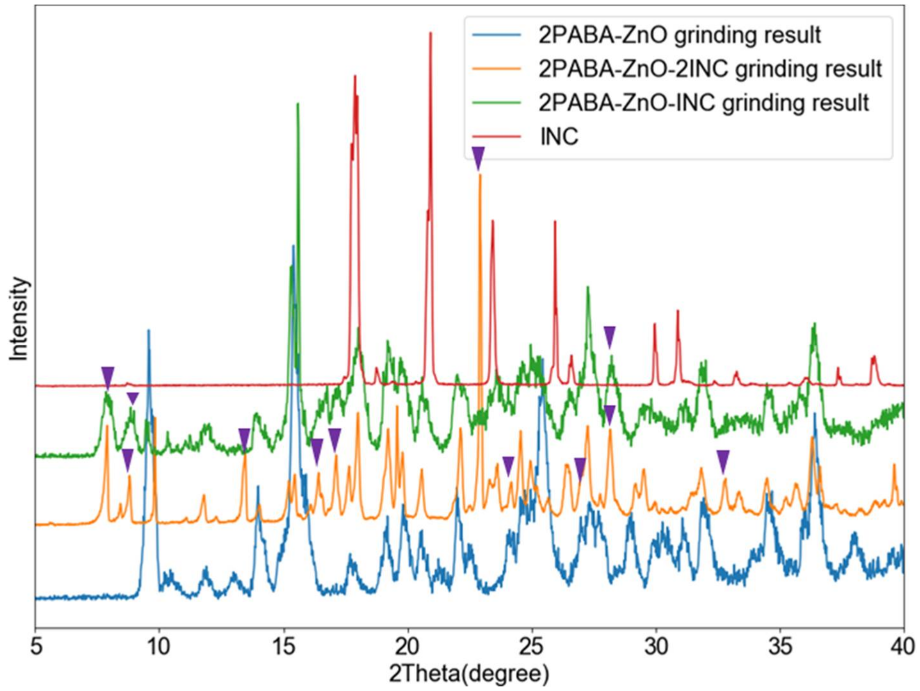

Figure S31. Comparison of PXRD patterns for INC (red), PABA-ZnO grinding result in 2:1 (blue) as well as PABA-ZnO-INC three-component grinding results in 2:1:1 (green) and 2:1:2 (orange) ratios.

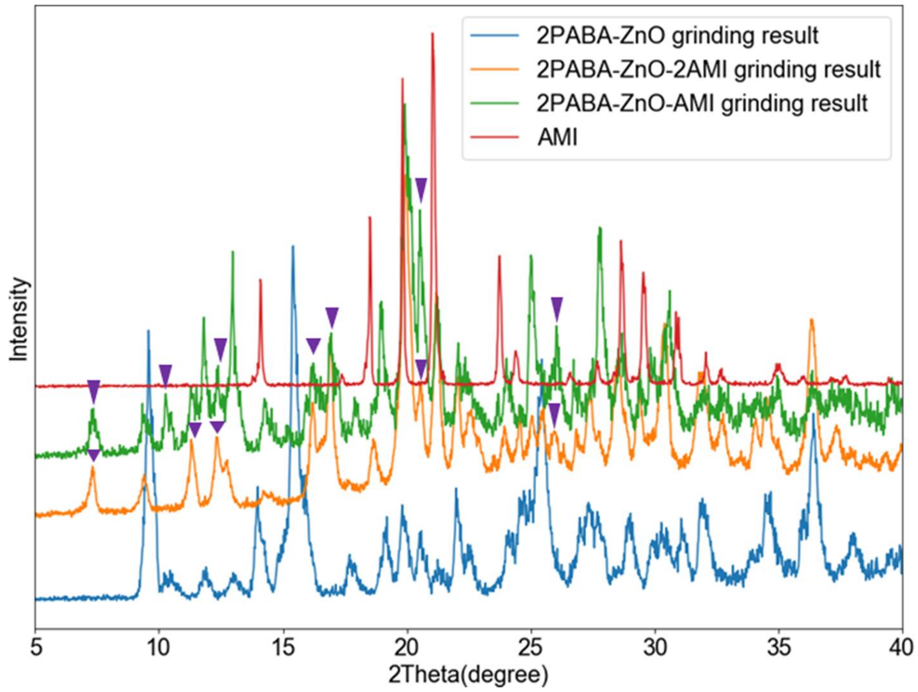

Figure S32. Comparison of PXRD patterns for AMI (red), PABA-ZnO grinding result in 2:1 (blue) as well as PABA-ZnO-AMI three-component grinding results in 2:1:1 (green) and 2:1:2 (orange) ratios.

4.1 VT-PXRD

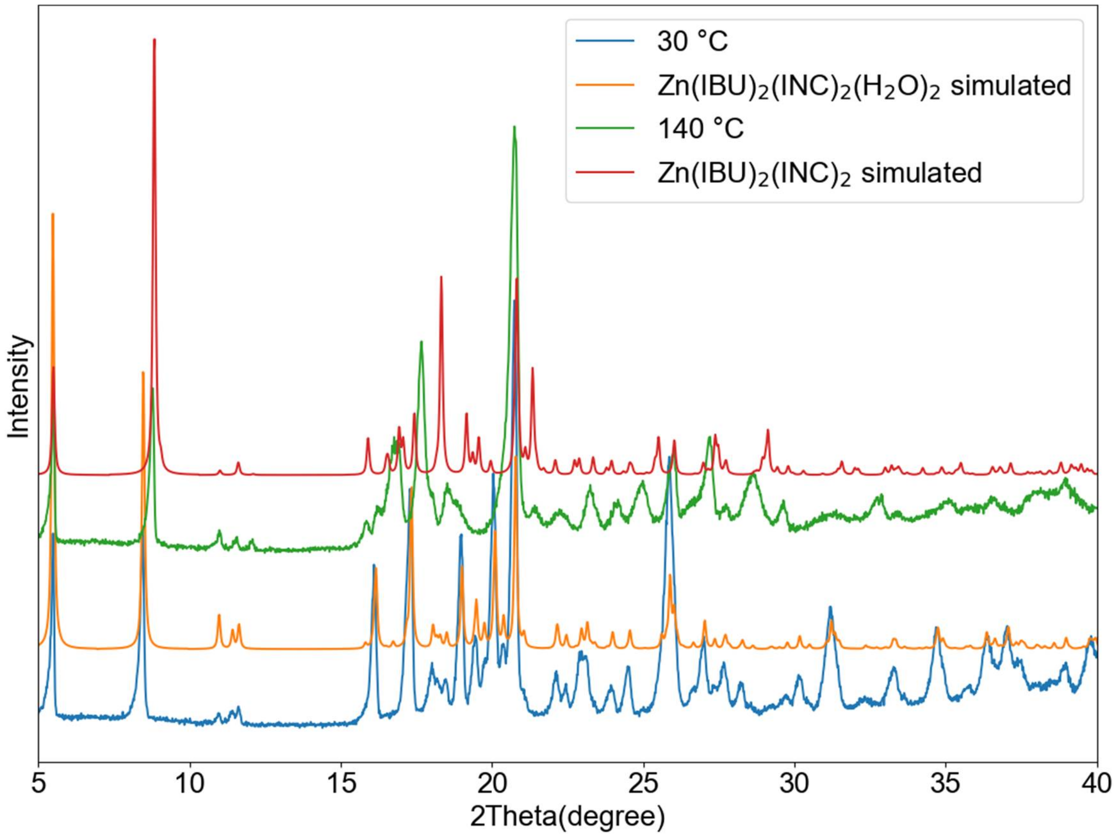

Figure S33. VT-PXRD analyses of $Zn(IBU)_2(INC)_2(H_2O)_2$ taken at 30 °C (blue) and 140 (green). The simulated PXRD patterns of the hydrated (orange) and the anhydrous (red) complexes are given for comparison.

5. Binary cocrystal screen

A binary cocrystal screen was performed between the organic compounds through methanol liquid assisted grinding. Most binary combinations also lead to cocrystal formation. However, this is not the case for eg. Aspirin-methylnocotinate.

	**Aspirin (ASP)**	**Ibuprofen (IBU)**	**4-aminobenzoic acid (PABA)**
**Methylnicotinate (MN)**	liquid	liquid	yes
**Nicotinamide (NC)**	yes	yes	yes
**Isonicotinamide (INC)**	yes	yes	yes
**Isoniazid (INZ)**	yes	no	yes
**Amifampridine (AMI)**	salt	liquid	yes
